# Autoimmune Retinopathy, Testing, and Its Controversies

**DOI:** 10.1007/s40135-021-00276-y

**Published:** 2021-10-08

**Authors:** Luiz Roisman, Julia Dutra Rossetto, Raquel Goldhardt

**Affiliations:** 1. Department of Ophthalmology and Visual Sciences, Federal University of São Paulo-UNIFESP/EPM; 2. Department of Ophthalmology – Hospital Federal da Lagoa – Rio de Janeiro; 3. Instituto de Puericultura e Pediatria Martagão Gesteira - Centro de Ciências da Saúde – Federal University of Rio de Janeiro; 4. Department of Ophthalmology, Bascom Palmer Eye Institute, University of Miami Miller School of Medicine, Miami, Florida

**Keywords:** Autoimmune retinopathy, cancer-associated retinopathy, melanoma-associated retinopathy, anti-retinal antibodies

## Abstract

**Purpose of review:**

The purpose of this revision is to sumarize the most important clinical features of the autoimune retinopathies (AIRs)

**Recent findings:**

AIRs are a group of inflammatory conditions affecting the retina characterized by progressive unexplained visual loss, abnormalities and contraction in visual fields, photoreceptor and electroretinographic dysfunction, and the presence of circulating anti-retinal antibodies. The pathogenesis of AIR remains unclear and various antiretinal antibodies have been associated to the disease. The diagnosis of AIR is based on a particular clinical presentation along with the detection of serum antiretinal antibodies. Numerous anti-inflammatory therapeutic alternatives have been described for the treatment of AIR, nevertheless there is no consensus on treatment protocol.

**Summary:**

Because of its association with different types of malignant tumors, the early diagnosis, multi-disciplinary approach and prompt treatment should be warranted.

## INTRODUCTION

Autoimmune retinopathy (AIR) is characterized by an inflammatory process affecting the retina that leads to photoreceptor dysfunction, scotoma, visual field defect, and acute or subacute vision loss in association with the presence of antiretinal antibodies (ARA).(1–4) AIR encompasses a spectrum of diseases, and has been divided in two main groups based on the trigger for the retinopathy, paraneoplastic and non-paraneoplastic retinopathy. The paraneoplastic autoimmune retinopathies are classically divided in cancer-associated retinopathy (CAR) and melanoma-associated retinopathy (MAR), although recently, bilateral diffuse uveal melanocytic proliferation (BDUMP) and vitelliform detachment were included in the AIR clinical spectrum.(1–4) A large group of autoimmune retinopathies that share clinical and immunological characteristics, but without an underlying malignant disease, are classified as presumed non-paraneoplastic autoimmune retinopathy. (1–4) The aim of this review is to improve the diagnostic capacity of this uncommon and clinically challenging retina disease.

## PATHOPHYSIOLOGY

There are certain proteins known to have an antigenic capability. Some of them are specific to the retina, such as recoverin, and others are also found in non-retinal tissues (for example, α-enolase). While recoverin and α-enolase are antigens widely associated with AIRs, others such as carbonic anhydrase, transducin-β, Tubby-like protein 1 (TULP1), neurofilament protein, heat-shock protein-70, photoreceptor-specific nuclear receptor, Müller cell-specific antigen, among others, have been suggested as possible retinal antigenic targets.(3, 5–8)

Evidence suggests that paraneoplastic AIRs can be triggered by molecular mimicry between tumor antigens and these retinal proteins.(5–7) Previous experiments have been conducted to elucidate the pathogenic role of antiretinal antibodies (ARA). In vitro studies have shown that recoverin and α-enolase induce apoptosis of retinal cells.(9) An in-vivo experiment showed that intravitreal injection of antibodies associated with MAR in monkey eyes caused electroretinographic changes similar to the disease in humans.(10) Despite this evidence, it is not yet clear why some patients with these antibodies develop AIR while others do not. ARAs can target any type of cell in the retina, including photoreceptors, ganglion cells or bipolar cells. However, the presence of these antibodies alone is not sufficient to diagnose AIR, as they can also be found in a variety of systemic autoimmune diseases, as well as in healthy patients.(1, 5, 11)

## EPIDEMIOLOGY

The non-paraneoplastic presentation of AIR is the most frequent. It usually affects individuals in the fifth or sixth decades of life that tend to be younger on average with female predominance compared to paraneoplastic AIR. (1–4)

Personal or family history of rheumatological or autoimmune diseases is common in non-paraneoplastic AIR.(1–4) CAR and BDUMP generally occurs in individuals in the fifth decade of life, with female predominance, although it can also affect young adults and elderly. Usually, retinal alterations precede neoplastic diagnosis. CAR has been associated with lung, prostate, colon, melanoma, hematologic malignancies, breast and gynecologic tumors (cervical cancer, endometrial carcinoma and uterine sarcoma).(1–4) Tumors of the ovary, lung, uterus, pancreas, colon and rectum, as well as cutaneous melanoma have been associated with BDUMP.(1, 2, 5) On the other hand, MAR generally occurs in patients with diagnosed cutaneous or acral melanoma.(1–4)

## CLINICAL FINDINGS

**Figure 1:**
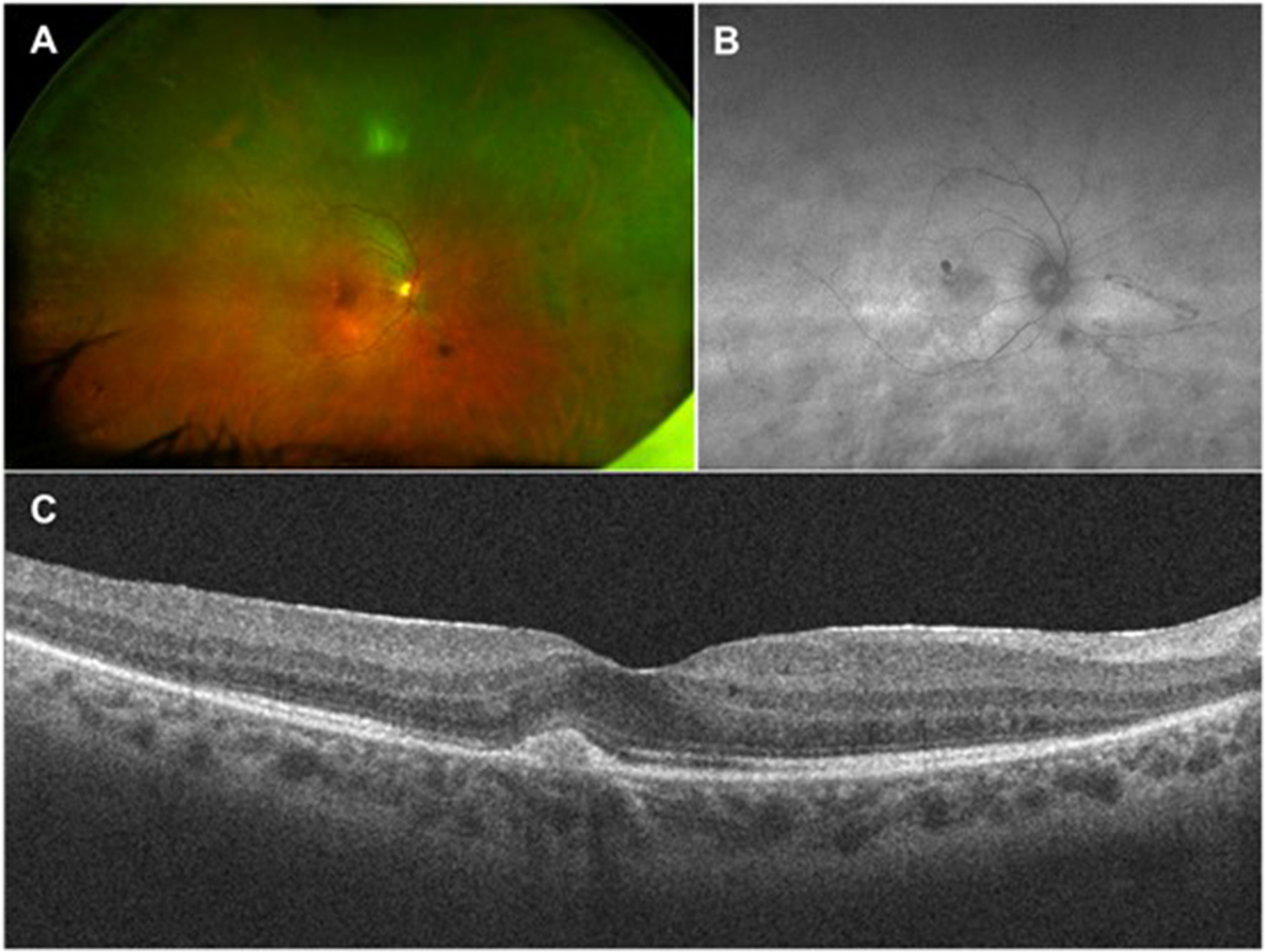
Multimodal imaging of the right eye of a 57 years old woman with suspected np-AIR after negative investigation of malignancy. Visual acuity was 20/25 on the right eye and 20/20 on the left eye, which had no abnormalities. (A) Wide field fundus picture showing mild vascular attenuation and pigmentary changes. (B) Fundus autofluorescence depicting a subtle hyperautofluorescent ring around the macula. (C) Optical coherence tomography revealing loss of the photoreceptor layer and thinning of the outer nuclear layer in the perifoveal region. This patient had been previously diagnosed with idiopathic choroidal neovascularization and treated with ranibizumabe 2 years prior to the AIR diagnosis. Its is notable a hyperreflective elevation of the foveal retinal pigment epithelium with no fluid.

The disease tends to present in one eye initially, then involving the contralateral eye within 4 days to 2 months. AIR usually presents as bilateral and asymmetric and more rarely, remain unilateral.(1–4, 7) Symptoms are typically subacute visual loss, visual field defect, scotomas, photopsia, nyctalopia, and dyschromatopsia. Fundoscopy may be normal or show discreet vascular attenuation, diffuse retinal atrophy, pigmentary changes, and optic nerve pallor (Figure 1).(1–4) Anterior chamber reaction or vitreous cellularity are usually absent or minimal. Retinal changes tend to appear early in CARs and late in MARs, especially when there are metastases.(1–5) Unlike typical AIR, with unremarkable fundus findings, BDUMP has well-defined clinical characteristics that include multiple and bilateral honeycomb fundus pattern displaying early hyperfluorescence on fluorescein angiography; the presence of multiple patchy melanocytic lesions with diffuse uveal thickening; exudative detachment of the retina; and early development of an opalescent cataract.(1–5)

## DIAGNOSIS

**Figure 2:**
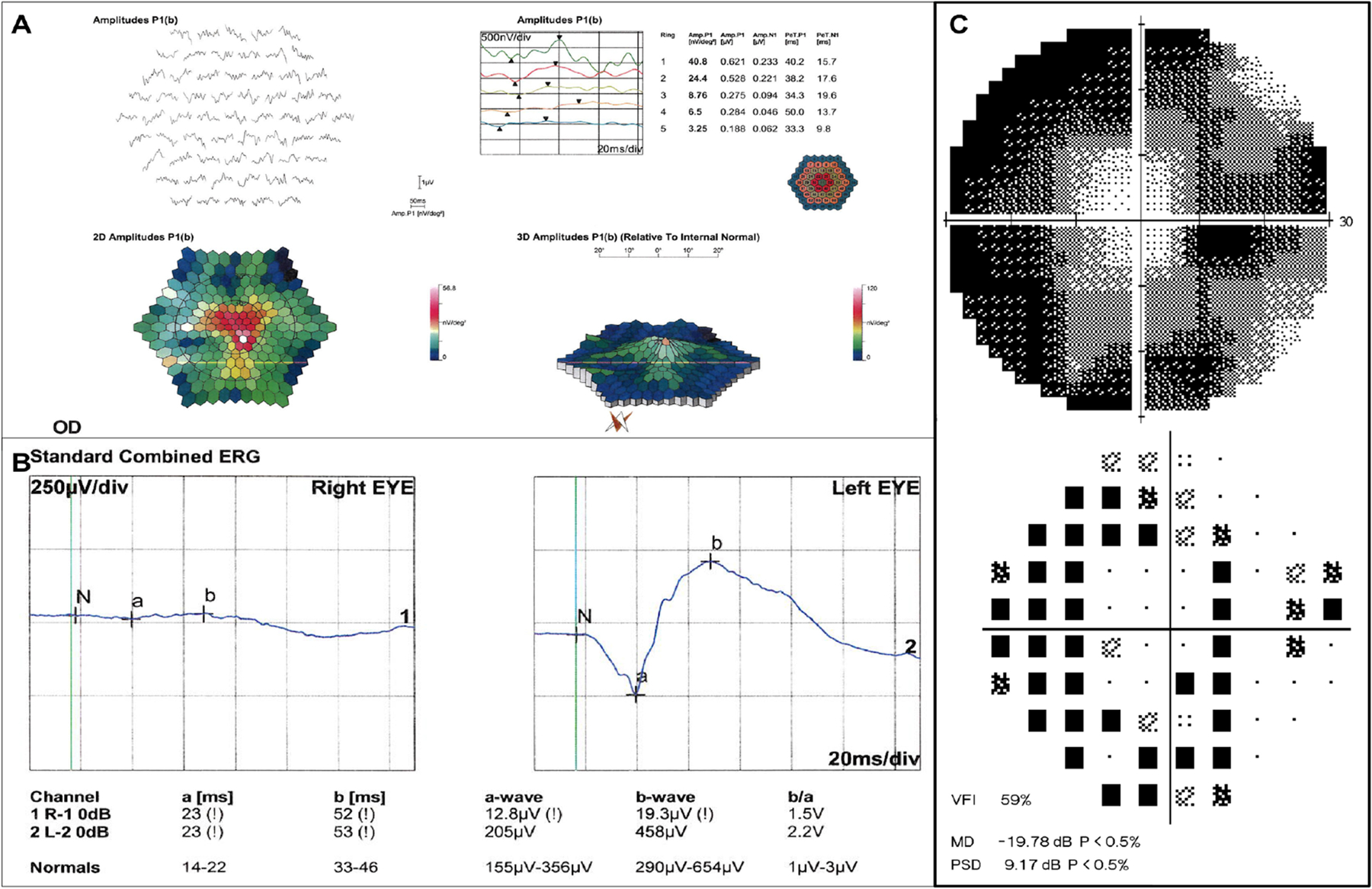
Functional evaluation of the same patient from figure 1. (A) Multifocal electroretinogram (ERG) showing loss of macular function. (B) Full-field ERG revealing global retinal dysfunction. (C) Visual field presenting with campimetric constriction.

The diagnosis or AIR starts with detection of visual field constriction, central or paracentral scotomas (Figure 2).(1–3) Visual field test should be repeated every 3–6 months and serves to monitor disease progression and response to treatment.

The electroretinogram (ERG) is one of the main diagnostic tests for AIRs because it presents specific abnormal findings depending on which cell is most affected (cone, rod or bipolar cells). The ERG classically shows global retinal dysfunction on multifocal and full field modalities, with reduced a and b waves (Figure 2). The ERG abnormalities may also vary between AIR spectrum diseases. Specifically, it usually depicts greater alteration in the cones in CAR and reduction of b waves in the full-field ERG in MAR.(1–3, 5, 12, 13) The electroretinographic findings may precede the fundus and anatomic changes demonstrated by optical coherence tomography (OCT).(1–3, 5, 12, 13) This is believed to happen because ARA initially decreases the function of neurosensory retina without leading to apoptosis.(1, 2, 5, 12, 13)

The OCT may reveal decreased macular thickness associated with outer retinal abnormalities such as loss or disruption of the photoreceptor layer and/or the outer nuclear layer (Figure 1).(14)

When AIR is accompanied by cystoid macular edema, fluorescein angiography (FA) may demonstrate leakage in the macular region. (1–3) FA is mostly performed to exclude other potential causes of visual loss that can present leakage and delayed staining, like vasculitis.

Fundus autofluorescence typically reveals a hyperautofluorescent ring in the parafoveal region, correspondent to the loss of inner segment and outer segment (IS/OS) junction and thinning of the outer nuclear layer on the OCT.(1, 2, 5, 14, 15).

## ANTI-RETINA AUTO-ANTIBODIES and controversies

Controversy surrounding the diagnosis of AIR continues because the differentiation between what is abnormal ARA (true positive) and a nonpathogenic baseline level of nonspecific circulating antibodies (false positive) involves repeat testing to monitor levels of ARA. It is important to consider that there could be variability in the detection and quantification of ARA between laboratories. Despite the uncertainties related to ARA, the presence of these antibodies is still considered essential for the diagnosis of AIR (Table 1). ARA have also been associated with specific disease and severity phenotypes. For example, positive anti-recoverin AIRs usually have a sudden, bilateral and symmetrical presentation, with severe central and peripheral vision loss, abnormal ERG and have a 90% association with cancer. Positive anti-enolase AIR, on the other hand, tend to have a subacute presentation, with variable and gradual vision loss, ERG varies from mild to severe changes depending on the level of cell disease and has a 40% association with cancer.(5, 6, 13, 16)

ARA can be detected using the Western-Blot (WB) method, immuno-histochemistry (IH) or ELISA test (enzyme-linked immunosorbent assay).(5, 17) Each method has advantages and disadvantages and each one of them can produce results that are influenced by various circumstances at each stage of the exam. That leads to a critical lack of standardization in AIR diagnosis. The WB allows the detection of ARAs by separating antigens from the retina based on molecular weight. Thus, this technique loses specificity since two different antigens can have similar dimensions, and be indistinguishable by this technique. IH involves testing the serum of a patient in whom AIR is suspected against a normal retina tissue from a human donor, ideally, or from a monkey or mouse; the sections are analyzed under a microscope to determine to which layer of the retina the antibodies binded. This is the great advantage of this method, the ability to determine the cell type that is affected. However, IH offers low sensitivity and is not capable to define the type of ARA involved.(1, 5, 13) Besides that, it requires high degree of uniformity, since each step of the testing including the type of fixating agent used or whether the sectioned tissue was fresh or frozen, can influence the result. The ELISA test analyzes several dilutions of the patient’s serum with specific retinal antigens and their binding is then measured by secondary antibodies. The main disadvantage of this method is the test’s specificity to each antigen, reducing the sensitivity to other antibodies that could be potentially involved in the retinal condition. Curiously, a study investigating the rate of agreement between two different laboratories running ARA assays, and found showed that their concurrence rate detecting any ARA was 60%. Among these, only 36% of the total cohort showed specific assay agreement for ARA.(17) And the total interobserver agreement was also very poor, with a kappa value of −0.13. The authors concluded that the lack of standardization of the tests led to dramatic variability in the laboratory detection of ARAs.

Although most experts believe the detection of ARA is a requirement for the diagnosis of AIR, others believe that they are possibly an epiphenomenon related to the disease, without direct pathogenicity.(8, 11, 18) Furthermore, some authors believe that the simple detection of the antibody is not sufficient for the diagnosis of AIR nor does it prove that that antibody found is pathogenic. (1) On the other hand, the absence of ARAs also does not rule out the diagnosis. In some cohorts, less than half of the suspected patients were positive for some antibody.(16)

## DIFFERENTIAL DIAGNOSIS

The differential diagnosis of AIR includes hereditary retinal diseases, various retinal degenerative disorders, toxic retinopathies and diseases of the spectrum of the white dots syndrome, multifocal choroiditis, acute zonal occult outer retinopathy (AZOOR), degenerative retinal disorders and uveitis. Besides the clinical aspect, ARA can also be positive in some inflammatory uveitis, such as Vogt-Koyanagi Harada syndrome, sympathetic ophthalmia, Behcet’s disease, and systemic lupus erythematosus, in infectious uveitis such as toxoplasmosis and syphilis, in degenerative diseases such as age-related macular degeneration, and in genetic conditions such as retinitis pigmentosa; this positivity reaches 90% of cases in some series.(1, 5, 16) Additionally, patients undergoing cancer monitoring, but without AIR, or even healthy individuals, can also test positive for ARAs, making it even more difficult to draw an ARA to AIR correlation. (1, 2)

## MANAGEMENT

Due to the systemic implications, it is important to differentiate paraneoplastic from non-paraneoplastic AIR with the guidance of an ocular oncologist. In paraneoplastic AIR, the reduction of the primary tumor with surgery, chemo or radiotherapy seems to be the best approach, although additional specific treatment to AIR may be eventually necessary.

Ocular treatment includes local and systemic immunosuppression. However, short-term ocular management can be initiated with intravitreal and sub-Tenons triamcinolone. This approach does not address the systemic condition, but help confirm the diagnosis before starting systemic long-term management with immunosuppressive therapy. Systemic or ocular corticosteroid therapy is used as a first-line treatment, followed by conventional immunosuppressants in the antimetabolite group, such as mycophenolate and azathioprine, and T-cell inhibitors, such as cyclosporine.(3, 5, 18, 19) Monoclonal antibodies, such as anti-CD20 (Rituximab), and intravenous immunoglobulin are options in resistant cases and, more rarely, plasmapheresis can also be used in selected cases.(3, 5, 18, 19)

Plasmapheresis seems to be more effective when used before visual loss, decreasing the circulating antibodies, and therefore the damage of the photoreceptors.(1, 3) Plasmapheresis has shown effective in treating patients with BDUMP.

Usually systemic treatment is utilized over at least one year, however, these different types of medications still produce variable therapeutic responses, even when compared observation alone.(3, 5, 18, 19) Also, no correlation between the type of ARA and the therapeutic response measured by visual acuity, electroretinographic and tomographic findings has been documented.

Studies have shown that anti-oxidants vitamins supplements such as beta-carotene (for non-smokers), vitamin C, vitamin E (for non-cardiac patients) and lutein seem to provide extra help against retinal degeneration.(4, 19)

Patient monitoring and follow up should include visual field and full field ERG every 3–6 months until stabilization is reached.(3, 5, 18, 19) The main goal is to stabilize visual loss.

## CONCLUSION

The mechanisms underlying AIR pathogenesis are incompletely understood. Clinical presentation is mild and diverse; temporal correlation to neoplastic diagnosis is not clear. Testing can help determine the presence of retinal autoantibodies, but laboratory methods are imprecise and the pathogenicity of ARA is uncertain. Thus, the diagnosis of AIR can be challenging and requires a thorough workup, and the importance of diligent inquiry and investigation to rule out hereditary retinal dystrophies and malignancy cannot be overstated. Treatment is another challenge since these are uncommon entities to which none of the available therapies showed unquestionable superiority, and most of the patients did not recover vision.

Further studies depicting cytokines and immunogenetic profiling of each AIR category are needed in order to establish the pathogenicity of ARA, allowing better standardization of treatment regimens as well more accurate assessment of clinical outcomes. Improving diagnostic tools and achieving consensus on specific management could not only preserve, but restore vision for these patients.

## Figures and Tables

**Table 1.** CAR - Cancer-associated retinopathy, MAR - melanoma-associated retinopathy, BDUMP - bilateral diffuse uveal melanocytic proliferation, AIR - Autoimmune retinopathy.

ANTIRETINAL ANTIBODIES COMMONLY ASSOCIATED WITH AUTOIMMUNE RETINOPATHIES (5, 13)	
CAR	Recoverin, α-enolase, Tubby-like protein 1 (TULP-1), heat shock protein 70 (HSP 70), Glyceraldehyde 3-phosphate dehydrogenase (GAPDH), anti-carbonic anhydrase II (AC II)
MAR	Transducin, arrestin, anti-bestrophin-1, melastatin 1, anti–aldolase A, anti–aldolase C, interphotoreceptor retinoid-binding protein (IRBP)
BDUMP	Recoverin, heat shock protein 70 (HSP 70)
AIR non paraneoplastic	Recoverin, α-enolase, arrestin, interphotoreceptor binding protein (IRBP), anti-Muller cell, anti-carbonic anhydrase II (AC II) and transducin
